# Critical Considerations for the Design of Multi-Organ Microphysiological Systems (MPS)

**DOI:** 10.3389/fcell.2021.721338

**Published:** 2021-09-09

**Authors:** Mridu Malik, Yang Yang, Parinaz Fathi, Gretchen J. Mahler, Mandy B. Esch

**Affiliations:** ^1^Department of Bioengineering, University of Maryland, College Park, College Park, MD, United States; ^2^Biophysical and Biomedical Measurement Group, Physical Measurement Laboratory, Microsystems and Nanotechnology Division, National Institute of Standards and Technology, Gaithersburg, MD, United States; ^3^Department of Chemical Engineering, University of Maryland, College Park, College Park, MD, United States; ^4^Department of Bioengineering, Materials Science and Engineering, and Beckman Institute, University of Illinois at Urbana-Champaign, Champaign, IL, United States; ^5^Department of Biomedical Engineering, Binghamton University, Binghamton, NY, United States

**Keywords:** MPS, body-on-a-chip, organ-on-a-chip, microfluidics, microphysiological systems

## Abstract

Identification and approval of new drugs for use in patients requires extensive preclinical studies and clinical trials. Preclinical studies rely on *in vitro* experiments and animal models of human diseases. The transferability of drug toxicity and efficacy estimates to humans from animal models is being called into question. Subsequent clinical studies often reveal lower than expected efficacy and higher drug toxicity in humans than that seen in animal models. Microphysiological systems (MPS), sometimes called organ or human-on-chip models, present a potential alternative to animal-based models used for drug toxicity screening. This review discusses multi-organ MPS that can be used to model diseases and test the efficacy and safety of drug candidates. The translation of an *in vivo* environment to an *in vitro* system requires physiologically relevant organ scaling, vascular dimensions, and appropriate flow rates. Even small changes in those parameters can alter the outcome of experiments conducted with MPS. With many MPS devices being developed, we have outlined some established standards for designing MPS devices and described techniques to validate the devices. A physiologically realistic mimic of the human body can help determine the dose response and toxicity effects of a new drug candidate with higher predictive power.

## Introduction

The development of new pharmaceuticals is time-intensive and expensive. A thorough assessment of a drug’s efficacy and safety must precede clinical testing in patients. Despite that rigorous evaluation, the success rate of clinical trials is low (Khalid et al., 2017). Preclinical tests conducted with animal models, despite their own benefits, often fail to show the same outcomes in human trials. It has been reported that of the drugs entering human clinical trials, 30% and 58% pass phase II and phase III, respectively (Thomas et al., 2016). The probability of drugs successfully moving from phase I to final approval is only about 9% (Thomas et al., 2016). Upon changing the patient selection process to have “selection biomarkers” as an inclusion or exclusion criteria, the probable success rate of drugs moving from phase I to approval stage increased to about 24% (Thomas et al., 2016). According to reports, out of the approved drugs in the market, 50% of the failures are due to unanticipated toxicity in patients (Tufts Center for the Study of Drug Development, 2013). Animal studies can falsely label a drug as “safe” or “toxic,” pointing to a need for more accurate alternatives to animal testing.

The development of human, cell-based multi-organ microphysiological systems (MPS) as a potential alternative to animal models has been pursued for over 20 years. An MPS is a miniature recapitulation of human organs fabricated *in vitro* to establish the functions of the organs. MPS can replicate the microarchitectures of human organs and mimic the human metabolism. The devices have inter-organ connections that allow drug metabolites generated in one organ or tissue to recirculate to all other organs within the system. In that way, drug metabolites can affect all tissues present in the system, offering the possibility of detecting secondary drug toxicity due to drug metabolites. MPS that are operated with human cells provide a unique opportunity for drugs to be tested in the context of human metabolism rather than that of an animal. The ability of MPS to detect the presence of toxic drug metabolites is an asset in improving the success rate of human clinical trials. The strive to achieve the same structural, functional, and biochemical attributes as a functioning human body makes MPS devices increasingly complex.

In this review, we discuss a variety of recently developed multi-organ MPS. We will also discuss opportunities to establish design criteria for MPS that offer a globally uniform and reproducible approach to experimentation with multi-organ MPS.

## Examples of MPS

Single-organ MPS have been constructed to serve as *in vitro* models for several different organs, such as the gastrointestinal (GI) tract (Kim et al., 2012; Lee et al., 2018), heart (Abulaiti et al., 2020), kidney (Wilmer et al., 2016), bladder (Sharma et al., 2021), liver (Deng et al., 2020), brain (Ndyabawe et al., 2021), lung (Huh et al., 2010), skin (Wufuer et al., 2016), skeletal muscle (Grosberg et al., 2012), bone (Bahmaee et al., 2020), vasculature (Wang et al., 2018), immune-system (Mitra et al., 2013), adipose tissue (Liu et al., 2019), and reproductive tract (Park et al., 2020). Here, we briefly discuss liver MPS, due to its significant role in drug metabolism, and a combination of single organ systems to establish more complex multi-organ MPS. We also highlight the development of MPS for three specific modules, namely the microvasculature, neuromuscular junctions, and the immune system, due to the recent interest in those modules, and their role in disease modeling.

### Single and Multi-Organ MPS

Since the liver performs some of the most important processes of human metabolism, much work has been done to develop advanced MPS devices to model the liver (Deng et al., 2019; Moradi et al., 2020). The use of 3D human cell cultures, as opposed to 2D cultures, provides an advantage to the MPS. Further, using non-parenchymal cells in co-culture with hepatocytes enhances (Kostadinova et al., 2013) and regulates drug metabolism. Prodanov et al. (2016) used a unique approach to build a complex, multi-cellular 3D liver-on-chip model. Stacking 2D monolayers of hepatocytes, endothelial cells and Kupffer cells with 3D dispersal of stellate cells in collagen yielded an organotypic *in vitro* model of the liver (Prodanov et al., 2016).

Adding recirculating fluidic flow to multi-cellular 3D liver cultures provides an opportunity for fast-paced nutrient and gas exchange that further enhances *in vitro* liver metabolism (Esch et al., 2015). Lasli et al. (2019) co-cultured HepG2 and human umbilical vein endothelial cells (HUVECs) to form spheroids, which were then moved onto a chip system to establish a steatosis disease-on-a-chip model. They used a network of interconnected hexagonal microwells to help assess the hepatic function in the model for the purpose of its applicability in drug toxicity testing at a later stage.

To mimic the oxygen gradient present in the *in vivo* liver, Kang et al. (2020) created an *in vitro* liver model with a microfluidic platform capable of generating gradients of varying oxygen concentrations. This hypoxia-on-a-chip platform was used to demonstrate the metabolic and genetic responses of hepatocytes to oxygen gradients (Kang et al., 2020). In-depth reviews of liver-on-a-chip models have recently been published by Deng et al. (2019) and Moradi et al. (2020).

Interconnections between two or more organs are built to analyze drug-drug, and drug-organ interactions, and secondary drug toxicity. Both the drug candidate and the information researchers need to gather with the MPS determine the number of organ chambers that are incorporated into an MPS. For example, the question of how much drug is available in the bloodstream after oral administration can be answered with a two-organ system that contains physiologically relevant amounts of blood surrogate. Several systems that mimic the first-pass metabolism and the subsequent bioavailability of drugs or toxicants in the systemic circulation have been developed since 2009. Mahler et al. (2009) presented a proof-of-concept gut-liver MPS that replicates *in vitro* the dose-dependent toxicity of acetaminophen on liver cells after metabolism by intestinal epithelium and liver. Another two-tissue system with GI-tract, liver, and “other organ” compartment designed by Esch et al. (2014) was used to investigate the effects of orally ingested polystyrene nanoparticles on the liver. The “gut-liver-other tissue” design used fluorescently labeled nanoparticles to track their travel across the gut barrier into the liver, and the eventual liver toxicity. Lee and Sung integrated a 3D gut model with a HepG2 cell layer, representing the liver, into a microfluidic device to determine the absorption and metabolism of digested lipids (Lee and Sung, 2018).

The integral role of the microbiome in gut function and homeostasis is represented in a gut-on-a-chip model using bacteria such as *Escherichia coli* (Tovaglieri et al., 2019), *Faecalibacterium prausnitzii* (Zhang J. et al., 2021), and *Lactobacillus rhamnosus* and *Bacteroides caccae* (Shah et al., 2016). The co-culture of epithelial cells with microbes to model microbe-host cell relationships provides a better understanding of their involvement in diseased and healthy intestinal conditions. Jalili-Firoozinezhad et al. (2019) successfully incorporated aerobic and anaerobic human gut microbiota on an intestinal chip with a hypoxia gradient. Compared to aerobic conditions, the inclusion of a hypoxia gradient improved the intestinal barrier function, and a more physiologically relevant level of microbial diversity was observed, allowing for microbiome-related therapeutic discovery and development in the future.

The true strength of MPS lies in their capacity to not only reveal primary drug action on human organs, but also secondary drug effects stemming from drug metabolites. Some of the first experiments aimed at testing this capability were conducted with three- and four-tissue devices. For example, a three-chamber MPS with cell lines representing liver, bone marrow, and tumor tissue was used to demonstrate the anticancer properties of 5-fluorouracil (5-FU) metabolites (Sung et al., 2010). The dynamics of the MPS were modeled using the same principles as physiologically based pharmacokinetic (PBPK) models to predict the effects of 5-FU itself and its metabolites on each organ cultured within the system (Sung et al., 2010).

Similarly, a four-organ device developed by Oleaga et al. (2016) showed that four tissues can survive and continue to function well for 14 days when cultured with a common cell culture medium. The recirculating flow of fluid in the device allowed the tested drugs to reach each tissue within the MPS and display multi-organ toxicity (Oleaga et al., 2016).

Miller and Schuler (2016) extended the concept of a multi-organ MPS to include up to 14 tissue chambers. The 14-chamber design was tested with tissues of five organs. Barrier tissues (skin, GI tract, and lung) and non-barrier tissues (fat, kidney, heart, adrenal glands, liver, spleen, pancreas, bone marrow, brain, muscle) were connected via fluidic channels that enable inter-organ interactions. This work demonstrated the feasibility of establishing a multi-organ MPS with more than four organ tissues and using it to measure basic cellular functions such as CYP450 enzyme activity, urea, and albumin synthesis, as well as tight junction maintenance.

While many MPS devices are closed microfluidic systems, an open multi-organ MPS was designed by Tsamandouras et al. (2017). As opposed to a closed system, an open system lacks one or more of the walls of the device, generating a liquid-air interface. The design by Tsamandouras et al. (2017) consists of a pump-based liver-gut MPS integrated on a polysulfone plastic plate, with recirculating flow of fluid. The system is based on the multi-well plate concept with a transwell-style gut MPS and 3D perfused liver MPS in combination with a mixing chamber. The initial design was later extended by Edington et al. (2018) to four, seven, and 10-organ MPS with internal recirculation and a pneumatic pump. A functional phenotypic co-culture was maintained for 2 weeks in the four-way MPS; this system was further extended to a seven-way and a 10-way MPS that sustained cell co-cultures for 3 weeks and 4 weeks, respectively.

### MPS for Modeling the Microvasculature

In the body, nutrients, oxygen, drugs, and other solutes must first cross the endothelial barrier before they can act on tissues. Because the endothelium is of broad interest—*in vitro* models have been used to model angiogenesis, thrombosis, and circulating cancer cell attachment—there are many microfluidic *in vitro* models of the vasculature available. Moses et al. (2020) discuss the advantages and disadvantages of some of the most recent vasculature-on-chip models.

The vasculature is particularly important for densely populated 3D tissues. Such tissues can develop a necrotic core due to the lack of nutrient and oxygen supply to the center. To construct tissues for use in MPS, the addition of blood vessels to 3D tissues helps better mimic the physiological rate at which drugs enter those tissues.

Miller et al. (2012) designed a biocompatible vasculature network where they first 3D printed mechanically stiff carbohydrate filaments (called carbohydrate glass) to form free-standing 3D fiber networks. The voids of those networks were filled with extracellular matrix (ECM) and cells and then cell culture medium was introduced. The medium dissolved the fibers, resulting in a channel network that was perfusable and that was surrounded by ECM and cells. When perfused with medium, cell function in the core of the tissue was healthier than in the non-perfused version.

Morgan et al. (2013) developed user-defined geometries of vascular networks using hydrogels to satisfy the needs of a variety of tissue models. Bischel et al. (2013) created 3D lumens through ECM hydrogels with different microchannel geometries and lined them with endothelial cells. A similar model with perfused functional vascular channels and multiple cell types was earlier developed by Lee et al. (2014) using 3D bioprinting technology. That work revealed the ability of vascular channels to present a functional physical barrier *in vitro.*

Vascular networks made from sacrificial material embedded in ECM or hydrogels hold a distinctive advantage over vascularized, microfluidic channels made of plastic, polydimethyl siloxane (PDMS), or other polymers because they allow cells to grow and remodel in the space between endothelial cells and ECM. A proper perfusion system clears secreted products and allows cell-cell interaction. Cochrane et al. (2019) present a detailed review of recent experimental vascular models and their importance in drug development.

While angiogenesis, the formation of new blood vessels from pre-existing vasculature, is important to normal growth and development *in vivo*, it is also necessary for tumor growth. In the absence of adequate vasculature, tumor cells become oxygen and nutrient-starved, which limits their survival and proliferation. This situation impacts the efficacy of radiation therapy (Gray et al., 1953), requiring 2.5–3-fold higher radiation doses (Joiner and Kogel van der, 2009). This knowledge has brought about the development of cancer therapeutics by inhibiting angiogenesis. Stroock and Fischbach (2010) discuss the development of microfluidic tumor models by combining vasculature and tumor tissue engineering with microfluidic technology to study antiangiogenic therapy. Patient-specific tumor microenvironments were also created by Truong et al. (2019) to analyze the tumor-stromal fibroblast crosstalk on a 3D microfluidic device. The use of patient-derived fibroblasts enabled a better understanding of their molecular and cellular influence on the tumor compared to generic, non-patient fibroblasts. Marturano-Kruik et al. (2018) developed a bone perivascular niche-on-a-chip model with stable vasculature within a 3D native bone matrix-on-a-chip. With this model, they achieved long-lasting, self-assembled vascular networks without the need for angiogenic factors to model the colonization of cancer cells in the bone.

The role of the immune system in tumor environment development is crucial and better understood (discussed in section “MPS for Modeling the Immune System”), however, the interaction of platelets with cancer cells remains to be better explored. Tumors can remodel vascular networks, leading to platelet extravasation (Schulte et al., 2011), which is difficult to model in animal models (Boussommier-Calleja et al., 2016). Saha et al. (2020) constructed an ovarian tumor-blood vessel-integrated organ-on-a-chip (OvCa-Chip) to study the time-dependent activity of endothelial cells in ovarian cancer and the eventual leakage of platelets through blood vessels. Using that model, they also tested a therapeutic strategy to prevent platelet extravasation in a cancer environment and validated their results with human ovarian cancer biopsy samples. With the rapid advancement in tumor-on-chip models, there have been numerous studies to incorporate the tumor microenvironment, discussed elsewhere in a comprehensive review on the topic (Wan et al., 2020).

Achieving a fully perfusable *in vitro* system within a 3D tissue is still challenging. Recent advances in pre-vascularizing tissues to achieve perfusion and engineering functional capillary beds to support the exchange between internal and external vessels (Zhang S. et al., 2021) can broaden the biomedical application of MPS even further.

### MPS for Modeling Neuromuscular Junctions

Neuromuscular junctions (NMJ) are modeled *in vitro* to explore clinical applications such as muscle and motor neuron-related diseases and spinal cord injuries. To successfully generate a functional NMJ, a combination of human motor neurons and skeletal muscle is established, which has been difficult to achieve perfectly *in vitro*. Although initial models involved simple culturing of dissociated motor neurons on a 2D layer of myotubes on a culture surface (Guo et al., 2011; Umbach et al., 2012), image analysis of such systems showed poor clustering of acetylcholine receptors and irregular co-localization of synaptic markers (Umbach et al., 2012). The MPS devices are aimed to model neurodegenerative disorders such as Alzheimer’s disease, amyotrophic lateral sclerosis (ALS), and tumors of the central nervous system like glioblastoma. To overcome the shortcomings of simple co-cultures, more sophisticated neuromuscular tissues are engineered *in vitro* using microfabricated devices with human stem cell-derived cell sources.

**FIGURE 1:**
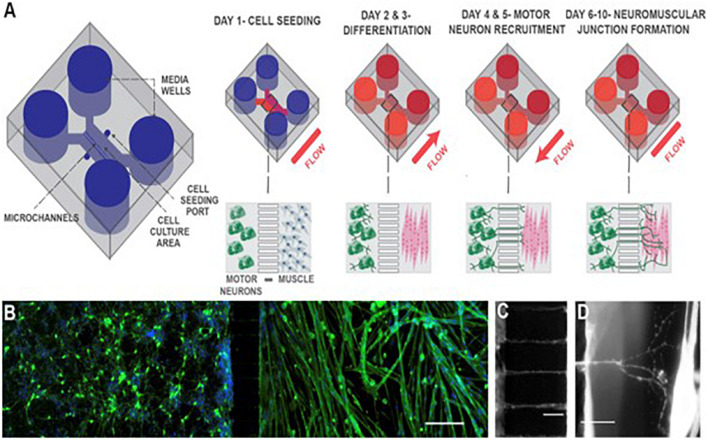
Schematic of neuromuscular junction microfluidic device. **(A)** Primary myoblasts and mouse embryonic stem cell-derived motor neurons were seeded in separate cell culture compartments in a microfluidic device via the cell seeding ports. Myoblasts were then differentiated (over 2 days), forming multinucleated myofibers. During this period, motor neurons were kept fluidically isolated. Motor neuron processes were then recruited across the microchannel, via a chemotactic gradient of gradient of glial cell-derived neurotrophic factor (GDNF) and brain-derived neurotrophic factor (BDNF) (days 4 and 5) generated by small but sustained fluid flow from the muscle to the motor neuron compartment. NMJs were allowed to mature until day 10, under static conditions. **(B)** Fluorescent image of microfluidic device showing motor neurons in the left-hand side compartment and muscle fibers in the right-hand side after 6 days of culture. Muscle fibers were stained for actin (green) and motor neurons were visualized by the Hb9:GFP reporter (green). Nuclei were stained with DAPI (blue). Scale bar represents 200 μm. **(C)** A close-up fluorescent image of neurite processes crossing the central microchannels. Scale bar represents 50 μm. **(D)** A close-up fluorescent image showing a neurite process extending, branching, and contacting a muscle fiber. Scale bar represents 50 μm. Reprinted from Mills et al. (2018) with permission from Elsevier.

Compartmentalized MPS culture motor neurons and engineered muscle bundles in individual compartments connected by axon-permissive channels. Using this approach, Mills et al. (2018) modeled NMJ formation and endocrine signaling in a microfluidic device, shown in Figure 1, using primary mouse myoblast-derived and embryonic stem cell-derived motor neurons. Santhanam et al. (2018) used the same approach to develop a functional NMJ system targeted to evaluate drug response and toxicity for ALS and other neurodegenerative diseases. The use of serum-free medium and stem cell-derived human myotubes and motor neurons in the device showed the possibility of developing patient-specific dose-response studies by incorporating patient-derived induced pluripotent stem cells (iPSCs). Osaki et al. (2018) used this model with iPSC-derived muscle cells and motor neurons to demonstrate reduced muscle atrophy and dysfunction in response to two ALS drugs, bosutinib, and rapamycin. The physiologically relevant layout of the compartmentalized MPS with human cells helps elucidate the pathogenesis of neuromuscular diseases and generate dose-response curves to potential drugs.

Smith et al. (2013) first proposed an *in vitro* system in 2013 to assess NMJ functionality and apply it to drug discovery research. They used the system to measure real-time responses of the cultured cells to chemical challenges, suggesting the possibilities of its application in preclinical drug screening. Guo et al. (2020) were able to use a compartmentalized NMJ model to reproduce clinical assays for assessing ALS deficits, but *in vitro*. This technology has set the path to use functional cell-based approaches, instead of preclinical models, for faster screening of novel therapies for ALS.

### MPS for Modeling the Immune System

One area that is gaining increasing interest in tissue-chip research is modeling components of the immune system. Many of these systems have been reviewed in detail elsewhere recently (Shanti et al., 2018; Kim et al., 2019; Sun et al., 2019; Greenlee and King, 2020; Maharjan et al., 2020), including discussions of microfluidic models of bone marrow, lymph nodes, the thymus gland, lymphatic cancer metastases, and inflammation. Human immune response in diseased conditions or during drug consumption is difficult to predict from an animal model. We will provide a brief overview of the latest developments in immune-on-chip models in the gut, liver, and tumor environment here.

Immune cell activation and the subsequent presentation of information to other cells plays a major role in generating an immune response to antigens. Dendritic cells typically capture antigens and subsequently migrate to lymph nodes to present antigens to T cells. Several on-chip immune studies have aimed to model immune cell migration in response to stimuli. Mitra et al. (2013) developed an *in vitro* model to study human cell line-derived dendritic cell migration with T cell activation. They demonstrated that mature dendritic cells migrate toward T cells in response to a CCL19 gradient, leading to T cell activation by the dendritic cells. Gopalakrishnan et al. (2015) developed a microfluidic device that incorporated an activator chamber to provide stimuli to cells in a migratory chamber. They demonstrated that immature dendritic cells from the migratory chamber moved toward the activator chamber when it contained a co-culture of dendritic cells and stimulated macrophages or mature dendritic cells (generated from C57Bl/6 bone marrow cells) stimulated with bacteria.

Although immune components such as macrophages have been included in a variety of single organ systems (Huh et al., 2010; Jones et al., 2012; Hamza and Irimia, 2015), their integration in a recirculating multiorgan MPS device is not advancing as rapidly. A recent article by Sasserath et al. (2020) presented the integration of a monocyte-derived cell line, THP-1, as a recirculating immune component in a multiorgan human model. The monocytes were added as part of the recirculating medium (blood surrogate) and allowed to flow through the three-organ system. The model showed activation of THP-1 cells in response to recirculation of amiodarone, a cardiotoxin tested in the study.

One aspect of the immune system that is of significance in microfluidic device development and has a potential of clinical importance is that of cancer-immune interactions (Parlato et al., 2021). Cancer cells exhibit immune-evasive behaviors that can limit the ability of the immune system to recognize and eliminate cancer cells. Microfluidic models have been employed to better understand these interactions. Aung et al. (2020) used a fluidic system incorporating a bilayer hydrogel to coculture human cell line derived breast cancer cells or spheroids, monocytes, and endothelial cells (HUVEC). They determined that T cell extravasation into the cancer-containing constructs occurred for both MCF7 and MDA-MB-231 breast cancer cell lines, and that extravasation was promoted by the presence of monocytes. This study demonstrated that various chemokines, such as those associated with hypoxia and loss of endothelial cell tight junction proteins, may play a role in T cell infiltration into tumors. Other studies have examined the interactions of cancer cells with lymphatic endothelial cells during metastasis formation. For example, Ayuso et al. (2020) developed a microfluidic model of tumor-lymphatic interactions and used it to demonstrate that cancer cells condition lymphatic endothelial cells to increase vessel permeability. Pisano et al. (2015) combined transwells with fluidic flow to evaluate the separate and combined effects of transmural and luminal flow on cancer cell invasion across an endothelial cell layer.

Microfluidic models have also been used to model inflammation (Irimia and Wang, 2018). Trapecar et al. (2020) modeled the gut-liver axis to develop a better understanding of the link between gut and liver diseases. They incorporated circulating Treg and Th17 cells within their system and found that during acute T cell-mediated inflammation, microbiome-derived short-chain fatty acids increased CD4+ T effector cell activation and liver injury. Shin and Kim (2018) developed a gut inflammation-on-a-chip model using a human intestinal epithelial cell line (Caco-2BBE) to understand the intercellular host-microbiome cross-talk that occurs in chemically-induced inflammation. Their studies identified barrier dysfunction as a critical trigger of intestinal inflammation and found that the efficacy of probiotics was altered when intestinal barrier function was disrupted.

Overall, recent attempts at microfluidic modeling of immune interactions have enabled not only the recapitulation of tissue structure and function but also the development of new hypotheses and understandings of biological phenomena that would not have been possible without the use of these microphysiological models.

## MPS Technologies

### Fluid Recirculation Methods

The transport system for the movement of nutrients, gases, and metabolites in an MPS is based on the techniques of microfluidics. As opposed to single perfusion systems, recirculation of fluid in the MPS allows transport of metabolites from one organ into other organ units, producing valuable pharmacokinetic information. Circulation of cell culture medium across tissues mimics the flow of blood in the human body. The cell culture medium is therefore sometimes referred to as blood surrogate.

Two or more organ chambers are connected via microchannels to establish communication between them in the MPS. Microchannel geometry and fluid circulation method are based on the specific nature of the tissue and organ-organ relationship of interest. For example, to mimic hemodynamic stimulation of cardiomyocytes, a peristaltic pump can be used with a cardiovascular chip to replicate the pressure-volume changes in the ventricle (Giridharan et al., 2010). Microchannel geometry is manipulated to increase nutrient and oxygen delivery. Ideally, the flow across tissues is maintained at near-physiological rates that replicate *in vivo* rates of exposure of tissues to nutrients and drugs. However, due to the micro-scale of the channels and chambers, many physical and chemical factors influence the flow of fluid. Capillary forces and flow rates are affected by the area and smoothness of the substrate. Lack of precision during the fabrication process can alter the working of the device to a great extent. Esch and Mahler discuss fabrication techniques suitable for building body-on-chip devices and how to experiment with them (Esch and Mahler, 2019).

Barrier tissue cells, such as gut cells, are generally seeded on a porous substrate where the top and the bottom portions have independent hollow fluidic channels to allow nutrient and drug transport across the barrier tissue. To further mimic the *in vivo* environment, specific organ-like forms, such as the villous shape of the intestine, can be designed using micro-molding techniques (Esch et al., 2012). Esch et al. (2012) used SU-8 layers to fabricate a microfluidic chamber and a porous membrane across the chamber. The structure could be made flat or into a 3D form by fabricating pillars of different sizes on the silicon surface (Esch et al., 2012).

**FIGURE 2:**
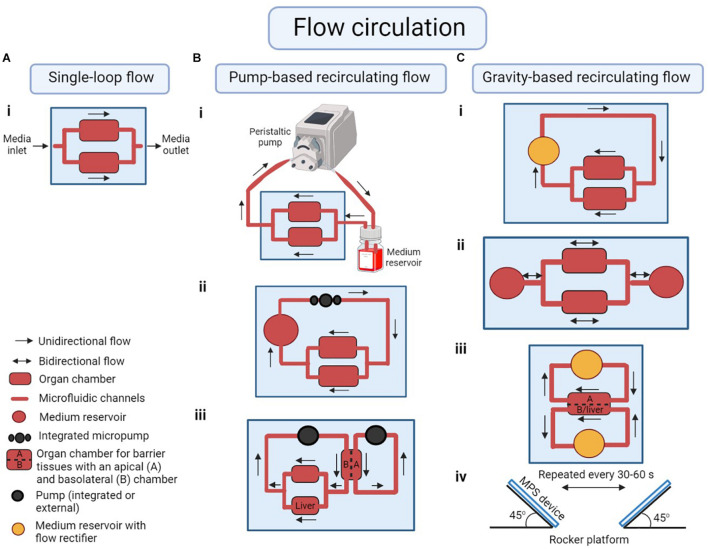
Examples of modes of flow circulation in a microphysiological system (MPS). **(A)** (i) Single-loop flow: unidirectional flow of medium across the organ chambers without recirculation. **(B)** Pump-based recirculating flow of medium using (i) an external peristaltic pump and medium reservoir to represent organs not cultured in any of the cell culture chambers in the system, (ii) an integrated on-chip pump, (iii) multiple integrated or external pumps to circulate medium through the apical side of a barrier tissue as well as the basolateral side of the tissue plus any other organ chambers (for example a liver-gastrointestinal tract MPS). **(C)** Gravity-based recirculating flow of medium following (i) unidirectional or (ii) bidirectional flow patterns with on-chip medium reservoirs. (iii) Medium reservoirs with flow rectifiers (or a valve) can be used in a liver-gastrointestinal tract model to regulate unidirectional flow in both apical and basolateral/liver chambers. (iv) The flow in pumpless systems is created by placing the device on a rocker platform at an angle, alternating the rocking motion every 30–60 s. Illustration is created using BioRender.com^‡^.

A factor that distinguishes different MPSs from each other is the method of fluid recirculation. Fluid recirculation in MPS is generally created by an active pumping mechanism, or by gravity-driven flow (Figure 2). External pumps such as peristaltic pumps or syringe pumps were the first to be used in MPS (Huh et al., 2010). Those pumps provide an easy way to create fluidic flow, but when used with multi-organ MPS, they require the flow to be split into multiple streams. Those streams must be regulated to create near-physiological flow rates for each organ chamber. Sin et al. (2004) developed a pump-driven three-compartment model on a 2.5 cm × 2.5 cm silicon chip with etched microchannels and chambers. They also included micropillar arrays to control the flow distribution in organ chambers (Sin et al., 2004). Zhang et al. (2009) combined micropillar arrays with hydrogel encapsulation to protect the cells from direct exposure to shear stress due to perfusion.

**FIGURE 3:**
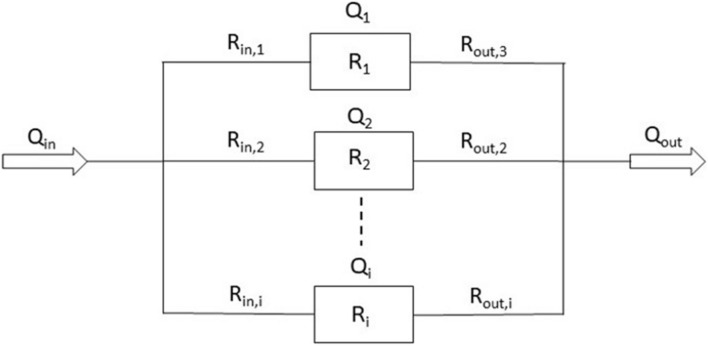
Schematic of the fluidic resistances of an MPS in which organ chambers are arranged in parallel (*Q*_*i*_ = flow rate through chamber i. Δ*p* = pressure drop across the channel/chamber segments. *R*_*in,i*_ = hydraulic resistances of inlet channel i. *R*_*i*_ = hydraulic resistance of organ chamber i. *R*_*out,i*_ = hydraulic resistance of outlet channel i.).

One way to achieve customized flow rates is to arrange all organ chambers in parallel so that the pressure drop across all chambers is the same (Figure 3). Then, by adjusting each organ chamber’s hydraulic resistance (including the inlet channel, organ chamber, and outlet channel), the flow in each chamber can be restricted to near-physiological values suitable for the tissue cultured within (Eq. 1 Bruus, 2008).

(1) $$Q_{\text{i}}=\frac{△p}{R_{\mathrm{in},i}+R_{\text{i}}+R_{\mathrm{out},i}}$$

Here, *Q*_*i*_ is the flow rate through chamber i in m^3^/s, Δ*p* is the pressure drop across the channel/chamber segments in pascal (Pa), and *R*_*in*__,i_, *R*_*i*_, *R*_*out,i*_ are the hydraulic resistance of inlet channel i, hydraulic resistance of organ chamber i, and hydraulic resistance of outlet channel i in Pa⋅s/m^3^, respectively. When designed with a particular length and depth, the widths of inlet and outlet channels lend themselves to adjusting the hydraulic resistances of each channel/chamber segment (Eq. 2; Bruus, 2008).

(2) $$R=\frac{12\eta L}{wh^{3}}*{\left[1-\frac{192h}{\pi^{5}w}\text{tanh}\left(\frac{\pi w}{2h}\right)\right]}^{-1}$$

Here, *R* is the resistance across the device in Pa⋅s/m^3^, *η* is the dynamic viscosity of the cell culture medium in Pa⋅s, *L* is the length of the channel in meters, *h* is the height of the channel in meters, and *w* is the width of the channel in meters. This equation is valid for channel dimensions that are such that *w* > *h*.

With more traditional devices being closed systems, Frey et al. (2014) developed a unique, open channel based MPS using an array of hanging drops as multiple parallel multi-organ microsystems. They used a peristaltic pump and open channels to form a microfluidic network between neighboring hanging drops (Frey et al., 2014).

However, because external pumps connect to MPS via tubing, the use of such pumps requires the presence of fluid volumes that create higher fluid-to-tissue ratios than would be physiological, often surpassing *in vivo* ranges.

Microfabricated on-board pumps, on the other hand, are integrated into the MPS itself and can help with reducing the volume of fluid needed to operate it. For example, Bowen and Scott incorporated a PDMS-based peristaltic pump and additional PDMS-based valves that recovered samples from the device (Bowen and Martin, 2010). Different placements and dimensions of the pump and valves were used to optimize flow rates. Schimek et al. (2013) used an on-board peristaltic micropump to operate a two-organ MPS. The pump produced pulsatile flow at a wide, adjustable range, creating physiological conditions for the growth of endothelial cells.

However, active pumping mechanisms can sometimes suffer from complications such as the formation of air bubbles, and possible leaking of medium.

To improve the reliability of fluidic flow, Sung et al. (2010) developed an MPS with gravity-driven flow, eliminating the need for a pump. The design consisted of multiple layers of PDMS with fluidic channels and organ chambers in the middle layers. To induce fluidic flow, the device was placed on a rocking platform that changed its angle periodically. In absence of a pump, all the fluidic components, i.e., microchambers, microchannels, and reservoirs were constructed on the chip without any external fluid or gas loops. Similarly, Oleaga et al. (2016) employed a range of platform tilt angles and oscillation times to achieve flow rates in the physiological range, and a human MPS company designed a variety of toxicology models that use gravity-based flow systems (Oleaga et al., 2016, 2018; McAleer et al., 2019a).

The flow Sung et al. (2010) and Oleaga et al. (2016) created in their systems is bidirectional, reversing direction periodically, albeit staying below a threshold shear rate. A tilt angle of 8° with two oscillations per minute resulted in maximum shear stress of 0.025 Pa (0.25 dynes/cm^2^) (Oleaga et al., 2016). Tissues that typically only experience low mechanical shear due to interstitial fluidic flow *in vivo*, are not disturbed by such low-grade, bidirectional shear (Esch et al., 2015).

Another advantage of gravity-driven flow is that MPS designs can be compact, eliminating long interconnecting channels from the designs as shown with the recently developed body cube (Chen et al., 2020), and thereby lowering the overall liquid volume needed to operate the systems to near-physiological blood surrogate levels (Chen et al., 2020). As opposed to a higher fluid volume that does not entirely represent the physiological scenario, such low liquid volume systems provide the ability to produce secondary metabolites at near-physiological concentrations.

However, when operated with small amounts of cell culture medium, gravity-driven, bidirectional flow can result in a small volume of cell culture medium that remains inside the cell culture chambers, not entering the liquid reservoirs on either side of the device, and not experiencing mixing with the remainder of the cell culture medium. That phenomenon can lead to poor oxygenation and a poor supply of nutrients in the middle of the cell culture chambers. When designing such bidirectional flow systems, care must be taken to operate the MPS with enough cell culture medium to achieve full recirculation of all cell culture medium.

Gravity-driven systems can also include passive, flow-rectifying elements that create unidirectional flow (Esch et al., 2016; Wang and Shuler, 2018; Yang et al., 2019; Fathi et al., 2020). Unidirectional flow is needed when tissues that experience high mechanical shear due to fluid flow *in vivo* are to be cultured within the system (most notably the endothelium). Yang et al. (2019) has found that such tissues thrive under unidirectional flow and display higher levels of inflammation under bidirectional flow, validating the use of unidirectional flow for producing functional endothelial cells.

Gravity-driven MPS often do not contain flow-regulating valves, thus the fluidic flow inside those systems is regulated by balancing the pressure drops and adjusting the hydraulic resistances as discussed for externally pumped systems that do not contain valves (Eq. 1 and 2).

### Integrated Sensors

Many MPS contain tissues and fluid volumes that represent between 1/10,000 and 1/250,000 of the organ and fluid volumes present in the human body. That scaling makes it possible to use reasonable numbers of cells to construct each tissue (~50,000–750,000 cells per tissue). But a drawback of the relatively small size is a limitation on the types of analysis that can be performed. Due to the small volumes of cell culture medium, often 1,000 μL or less, retrieval of fluid samples from the system for external analysis, without disturbing its dynamics, is a challenge. Nevertheless, analysis of tissue health is often accomplished by measuring concentrations of soluble proteins such as albumin, urea, and inflammation markers in the cell culture medium taken from the MPS at various times. Microfabricated, system-integrated sensors that measure the physiological response of tissues to chemicals or drugs can provide for such analysis without distorting metabolite and marker concentrations.

Owing to the significant role in drug metabolism, several types of sensors were developed to evaluate the status of liver tissue. For example, aptamer-based electrochemical sensors have been used to detect liver-generated proteins through protein binding to the electrode and the subsequent change in electrode redox properties. Son et al. (2017) added a sensing channel adjacent to the liver chamber, separated by a polyethylene glycol (PEG) hydrogel barrier. Detection occurs in the sensing channel via a standard fluorescence-based antibody sandwich assay (i.e., primary antibody-coated, non-fluorescent capture beads and secondary antibody-coated, fluorescent detection beads). Upon diffusion of proteins of interest into the sensing channel and binding to the capture beads, the fluorescent beads bind and aggregate on the surface of the capture bead; the resulting fluorescence intensity is detected through imaging.

Bavli et al. (2016) included luminescence and enzyme-based sensors to measure oxygen and glucose/lactose concentrations in the system. Monitoring those small molecules provides vital information about the status of metabolism inside the system. Similarly, Sin et al. (2004) developed a dissolved oxygen sensor as a proof-of-concept model to measure gas exchange in an MPS. Instead of the widely used Clark electrodes, which consume oxygen in order to detect it, Sin et al. (2004) integrated a non-invasive, fluorescence-based oxygen-sensing system and confirmed that oxygen exchange is adequate inside the device. The oxygen sensing was based on phase-sensitive detection using a ruthenium complex as a fluorescent probe. A calibration curve of observed phase angles and known partial oxygen concentrations (*p*O_2_) was used to find the oxygen exchange in the system with respect to the changes measured in phase angle.

**FIGURE 4:**
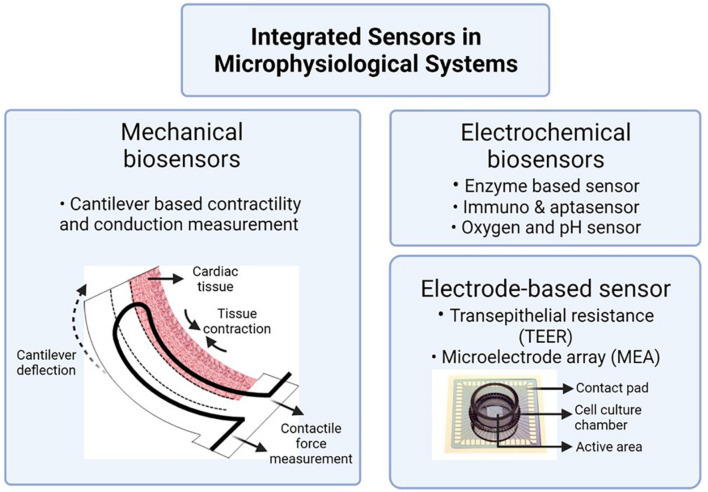
Integrated sensors to assess tissue health in MPS. Image is created using BioRender.com^‡^.

Cardiac models are typically combined with added equipment to monitor heart cell function. Contractile forces of heart muscle cells can be assessed using a cantilever deflection system. Cantilever deflection is used to record changes in cardiac and skeletal muscle contractile forces in response to drug exposure (Smith et al., 2014; Oleaga et al., 2016; Sasserath et al., 2020). Cantilevers are microfabricated from silicon-on-insulator wafers on the device layer and with a window underneath that allows access to laser light. Contraction of cardiomyocytes or skeletal cells seeded on top of the cantilevers is measured from laser deflection in response to the cantilever bending during muscle movement. Figure 4 depicts the working of a cantilever along with other commonly integrated sensors in MPS.

Electrical activity and beat frequency of cardiomyocytes and electrical activity of skeletal muscle cells can be measured by growing the cells on microelectrode arrays (MEAs). In 1980, Pine (1980) used the MEAs to record action potentials of dissociated neuron culture from neonatal rats. MEAs remain the gold standard for electrophysiological assessment of cardiomyocytes *in vitro*. While a standard MEA has about 60 electrodes, Sasserath et al. (2020) used a customized MEA with 10 recording/stimulating electrodes, each with a diameter of 200 μm, and one grounding electrode of diameter 2,000 μm. The electrodes are linked to a stimulus generator to induce electrical stimulation to the cells.

Kanagasabapathi et al. (2013) adhered PDMS-based microchannels on commercially available MEAs to investigate the effects of tetrodotoxin on a model of the central nervous system. Over the years, electrode-cell conformity has improved to achieve a better signal-to-noise ratio. One such example is the tower design of a 3D hippocampal network by Rowe et al. (2007), which was later used by Musick et al. (2009) to record cortical neural electrical activity.

When using barrier tissues in MPS, assessment of the tissue’s barrier integrity holds immense significance. Integration of electrodes to measure transendothelial and transepithelial electrical resistance (TEER) helps evaluate that barrier function. For example, the endothelial lining of blood vessels in the brain (blood brain barrier, BBB) plays a crucial role in regulating and protecting the central nervous system. Douville et al. (2010) first reported the use of TEER electrodes to assess the integrity of the BBB in real-time. A combination of different electrode-based sensing techniques, both MEA and TEER electrodes, was used by Maoz et al. (2017) to validate cardiomyocyte beat-rates and endothelial barrier function in response to inflammatory cytokine and isoproterenol.

In addition to models of the blood brain barrier, TEER is also used in vascular, gut (Esch et al., 2016), lung (Walter et al., 2016), skin (Sriram et al., 2018), and kidney (Douville et al., 2010) models. In most cases, Ag/AgCl electrodes are integrated into the cell culture platform to read TEER values. TEER measurements are heavily dependent on electrode placement, cell layers, and membrane geometry. In the case of MPS, the placement of organ chambers (e.g., tower or adjacent), temperature, and cell culture conditions can also change the TEER measurement (Srinivasan et al., 2015). While TEER values are widely studied and standardized for transwell cultures, more work needs to be done to have comparable data across different microphysiological systems.

### MPS for Acute and Long-Term Toxicity Measurements

The length of operation of MPS is based on the purpose of the study to be conducted. Experiments designed to test acute toxicity can be accomplished with a short drug exposure time of under 48–72 h. An example of an acute response study is that published by Oleaga et al. (2016) who challenged their four-organ, pumpless MPS with five different test compounds for 48 h. Toxicity of each drug on liver, muscle, brain, and heart was observed during that exposure time. Chen et al. (2020) reported the development of a simple yet reliable MPS device for short-term operation (up to 72 h) with near-physiological amounts of fluid volume in the system. The cell viability and liver function assays showed a successful short-term operation of the MPS with a near-physiological amount of blood surrogate (Chen et al., 2020).

Identifying the effects of chronic drug exposure, however, requires MPS operation for longer than 72 h. Longer operation of the device can be challenging because it requires continued tissue function when tissues are cultured with a common cell culture medium that addresses the needs of all tissues, but may no longer be specialized for any tissue in particular. The increased experimental time also means functional and morphological assessment of the cells in the model for a longer duration. The maintenance of tissue integrity with continued assessment can present a challenge of its own. Nutrients are consumed, and cellular waste products accumulate over time. In addition, the dynamic nature of MPS results in an even higher rate of metabolite synthesis and secretion compared to static cultures and even more so in co-cultures (Guzzardi et al., 2009). A relatively simple way to solve this problem is to replace part of the consumed cell culture medium with fresh medium every day. Using this technique, MPS have been operated for 7 days (Oleaga et al., 2018), 14 days (Oleaga et al., 2016), and for up to 28 days (Maschmeyer et al., 2015; Xiao et al., 2017; Oleaga et al., 2019). A period of 28 days is the minimum duration to evaluate repeat dose toxicity in animal models, leading to the use of this long-term *in vitro* time frame. Similar to removing cell culture medium for the purpose of tissue-health assessment, a drawback of a daily medium exchange is that concentration profiles of both drug and drug metabolites may no longer entirely reflect their natural, physiological profiles.

While several models have been developed that can maintain the cultures for a long period, exposure to the drug over the entire duration has not been entirely achieved. Wagner et al. (2013) exposed a liver-skin MPS to troglitazone drug repeatedly at 12 h intervals for 7 days to test the system for repeated dose substance exposure. The group observed an increase in cytochrome P450 3A4 enzyme level at day 7 of exposure compared to the controls. A detailed review by Zhao et al. (2019) documents the advancements in multi-organs MPS development for long-term exposure investigations. While current long-term exposure analysis is still primarily performed in animals, the steady development of MPS is moving toward substituting animal-based chronic exposure testing as well.

## Design Principles for MPS

To date, many single-organ and multi-organ MPS that focus on different aspects of device performance and drug testing have been published. Despite a considerable variety in designs, certain features of MPS have proven useful over time. Such features ensure the feasibility, reproducibility, and physiological relevance of the devices. Knowing the design criteria of an MPS can provide insight into its possible applicability to another group’s work. Here we discuss some design components that are often utilized in MPS development. Of those components, we discuss three essential design elements (Table 1) that we think researchers can use to compare their MPS with those of others.

**TABLE 1:** Factors considered for the design of MPS.

Design criteria	Suggested range	Considerations
Scaling factor	50,000–100,000	Impacts the size of the system. A higher scaling factor will generate a larger system, increasing the fluid volume, and the number of cells needed to reach functional organ volume ratios, thus making the device more expensive. A lower scaling factor will generate a smaller system, with small volume of blood surrogate but also possibly cause difficulty in system operation.
Liquid volume	1/10,000–1/250,000 of the *in vivo* organ and fluid volume	Higher fluid volume can dilute the metabolites produced in the device, making measurements of metabolite toxicity less relevant to what occurs *in vivo*.
Fluid-to-cell ratio in tissues	10^3^–10^6^ cells based on scaffold area	Value is based on the physiological cell density, functional organ volume (eliminate the volume occupied by the scaffold material) and seeding area.

### Functional Organ Volumes

Multi-organ MPS are designed to replicate key aspects of human physiology. Indeed, the power of MPS lies in the devices’ potential to recreate human drug metabolism with drug and metabolite concentration profiles that can closely mimic those occurring *in vivo*. Achieving that outcome requires MPS to be designed with a few yet important design criteria in mind.

First, as suggested by Wikswo (2014) in their seminal paper, an ideal MPS reproduces the relationships of functional *in vivo* organ volumes. The underlying assumption is that the amount of drug that is absorbed, consumed, and/or metabolized *in vivo* depends on the activity of available enzymes, which, in an MPS, depends on both, the quality of tissues and tissue volumes (Eqs. 3-1, 3-2 Bruus, 2008). The drug metabolism rate is directly proportional to the organ volume where metabolism takes place. When considering the volumes of tissues, particular attention must be given to the function of different tissues within an organ. To correctly relate functional organ volumes *in vitro*, each organ of the human body must be considered in terms of its function with regards to drug absorption, metabolism, and elimination, and each tissue should be scaled to keep the physiological volume ratios intact (Wikswo, 2014).

(3-1) $$V_{\text{o}}\frac{\text{d}C_{\text{o}}}{\text{d}t}=Q_{\text{O}}C_{\text{b}}-\frac{Q_{\text{o}}C_{\text{o}}}{P_{\text{o}}}-CL_{\text{int}}fC_{\text{o}}$$

(3-2) $$V_{\text{o}}\frac{\text{d}C_{\text{o}}}{\text{d}t}=Q_{\text{O}}C_{\text{b}}-\frac{Q_{\text{o}}C_{\text{o}}}{P_{\text{o}}}-V_{\text{o}}\sum \frac{V_{\text{m}}fC_{\text{o}}}{K_{\text{m}}+fC_{\text{o}}}$$

In Equation 3-1, *V*_o_ is the volume of an organ in milliliters, *t* is time in minutes, *C*_b_ is the concentration of drug in blood in mmol/mL, *C*_o_ is the concentration of drug in the organ in mmol/mL, *Q*_o_ is the blood flow rate going through the organ in mL/min, *P*_o_ is the partition coefficient of drug in the organ, *CL*_int_ is the intrinsic clearance of the drug in the organ in mL/min and *f* is the unbound fraction of drug. For the metabolism of drugs in the organ that follow Michaelis-Menten kinetics, the equation could be written as Eq. 3-2. Here, *V*_m_ is the maximum reaction rate of the enzyme in mmol/(mL⋅min), and *K*_m_ is the Michaelis constant of the enzyme mmol/mL.

When replicating functional units of an organ, it is also important to consider the source of cells used to create the tissue mimic and the level of enzyme activity within those cells. The goal is to achieve drug conversion rates that are close to those *in vivo* (Eqs. 3-1 and 3-2). One of the most important considerations, for example, is the design of the liver tissue. The closer the metabolic activity of liver cells inside the MPS matches that of liver cells *in vivo*, the better the liver will replicate *in vivo* concentrations of drug metabolites. However, *in vitro* enzyme activities rarely match *in vivo* activities, and an extra volume of cells may be needed to reach a certain *in vivo* functional organ volume.

Due to the difference in *in vitro* and *in vivo* metabolic activity, an analysis of the enzymatic activity involved in the metabolic pathway is a critical first step to ascertain the proper functioning of an MPS.

### Blood Surrogate Volume and Formulation

Culture conditions for multi-organ MPS differ considerably from single-organ MPS due to the many cell growth requirements of the different tissues. The local *in vivo* environments of human organs provide certain biochemical characteristics such as growth factors and cytokines. Those features have been shown to have a significant influence on the development and function of organs, thus making it good practice to include them in MPS as well (Wang et al., 2020).

In *in vitro* conditions, blood surrogate is a substitute for blood, which is why it must recapitulate human physiological oxygen transport characteristics. In addition, the common cell culture medium that recirculates among all organ chambers must be able to support the phenotypic and functional requirements of all those tissues. Even though there is currently no universal serum-free medium, trying different formulations of a minimal medium can help determine the right medium. To start, a base medium can be created using an already existing cell-type specific medium or choosing a medium suitable for a high demanding cell line, followed by the addition of supplements or growth factors. For example, because of the liver’s high metabolic demand and its integral role in drug metabolism, the formulation of a common MPS cell culture medium is often based on liver cell culture medium (Sung et al., 2019). Additional growth factors that support other cell types can be added to that base medium. Zhang et al. (2009) were able to create a blood surrogate that supported four cell types.

Alternately, to ensure optimum culture conditions for all cell types present in the MPS, it is good practice to perform a growth assessment in the intended medium and adjust the formulation based on the cells’ responses.

Fluid content in the MPS device is divided into the circulating volume of blood surrogate in the system (all liquid or cell culture medium that resides in fluidic channels, reservoirs, sensor cavities, or that simply flows across a tissue on its surface) and the interstitial liquid volume (liquid or cell culture medium that is present within the organ chamber volume that is occupied by the tissue volume, i.e., the scaffold or matrix and the actual cells). The volume of cell culture medium that functions as blood surrogate within an MPS must be carefully considered. Both the amount of interstitial liquid and the volume of blood surrogate should ideally be close to physiological levels. To achieve a reasonable number of 50,000–750,000 cells per tissue, the *in vitro* tissue and fluid volume is kept between 1/10,000 and 1/250,000 of the *in vivo* organ and fluid volume. Reporting the liquid volumes used with an MPS can help when comparing data obtained with different designs, thus becoming a vital evaluation factor in our opinion.

**FIGURE 5:**
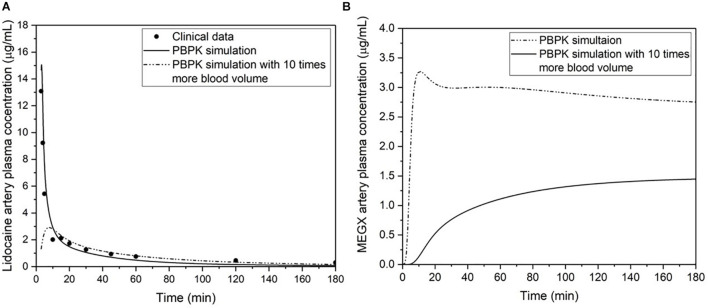
**(A)** The PBPK simulation and clinical data of lidocaine artery plasma concentration change over time after an intravenous administration of 3 mg/kg body weight (Tucker and Boas, 1971). **(B)** Comparison of two simulations for the artery plasma concentration change of monoethylglycinexylidide (MEGX, a metabolite of lidocaine) after an intravenous administration of 3 mg/kg body weight, and using published parameters for MEGX unbound fraction (Feely and Grimm, 1991), partition coefficient (Dillane and Finucane, 2010), and *in vitro* liver kinetics (Bargetzi et al., 1989). One simulation is modeled with physiological parameters, and the other is modeled with 10 times more blood volume while keeping all the other parameters equal and at physiological values.

The blood surrogate volume for an MPS can be calculated by dividing the typical volume of blood in a human body by the scaling factor used to calculate the functional organ volumes for all other organs. Since the blood surrogate collects within the medium reservoirs and gets redistributed from there, it pools all metabolites from the different organ chambers. An increase in blood surrogate volume larger than normal physiological volumes will dilute drug metabolites, leading to difficulty in assessing the drug efficacy or potential toxicity. To support this statement, we ran a PBPK simulation for clinical data of lidocaine artery plasma concentration change over time after an intravenous administration of 3 mg/kg body weight (Tucker and Boas, 1971; Figure 5). Upon increasing the blood volume by 10 times, the concentration of monoethylglycinexylidide (MEGX, a metabolite of lidocaine) in plasma was seen to be lowered. This reiterates the importance of following physiological values of blood volume *in vitro* to accurately determine the concentration of toxicant in the system.

The need to regulate the blood surrogate volume so it remains close to physiological levels is solved by using a scaling factor. It determines the physical size of the system and how much blood surrogate volume it should be operated with. Designing an MPS system with a physiological equivalent of *in vivo* blood volume often translates to a small volume of culture medium 60–300 μL, which makes it difficult to operate the MPS. Scaling factor is one of the most important characteristics of an MPS thus qualifying as second of the three important criteria for MPS evaluation.

Currently, MPS scaling factors range between 50,000 and 400,000. Scaling factors between 50,000 and 100,000 are likely the most useful because smaller systems will require very small volumes of blood surrogate, making it difficult to operate the system and successfully sample medium for drug concentration analyses. If the system is large, all organ mimics will require many cells to replicate functional organ volume ratios. This type of device may become expensive when working with tissues constructed from primary cells or stem cell sources.

The body-in-a-cube design by Chen et al. (2020) uses 80 μL of blood surrogate, 1/73,000 of the blood volume (with a scaling factor of 73,000), to achieve near-physiological volumes. The group was able to demonstrate successful functioning of the four-organ MPS with this small circulating volume of fluid for 72 h.

### Fluid-to-Cell-Ratios in Tissues and Cell Culture

As mentioned in section “Blood Surrogate Volume and Formulation,” the amount of fluid within an MPS is an important determinant of how well the sensors in the devices can detect the presence of toxic metabolites. Too much liquid will dilute metabolites and make their effects on tissues less pronounced. The amount of fluid within each tissue, i.e., the amount of liquid that represents the interstitial liquid plays a similar role as the blood surrogate.

To construct tissues for MPS, cells are typically seeded onto scaffolds or into hydrogels and allowed to mature before exposing the MPS to drugs or chemicals. Natural biomaterials such as collagen and fibrin (protein-based), hyaluronic acid and cellulose (polysaccharide-based), and decellularized ECM can support cell adhesion and growth because they are compositionally consistent from batch to batch.

Since the response of a drug or chemical in the human body is highly dependent on the local environment of the body, it is imperative to replicate the *in vivo* environment as closely as possible. The cell density for individual organs and the seeding ratio of cells making up a particular tissue are based on the physiological density of cells in the human body. A typical organ in the human body is about 100–500 mL in size with about 10^9^ cells per organ, with some organs having more and some having slightly less (McClelland et al., 2012). However, to make a physiologically relevant model, the average cell number in an *in vitro* system is based on the dimensions of the scaffold and ranges from 10^3^ to 10^6^ cells. A higher cell density than this can result in overcrowding of the attachment surface, potentially leading to cell mortality and irregular metabolite buildup. This difference between the *in vivo* and *in vitro* cell densities needs to be considered when calculating functional organ volumes. To obtain more accurate *in vitro* values, the volume that is occupied by cell culture scaffolding material could be subtracted from the overall chamber volume (Chen et al., 2020).

Currently, most 3D cell cultures do not yet replicate *in vivo* cell densities, and therefore contain larger amounts of interstitial liquid than would be physiological. Maintaining an optimum cell count and continuous fluid flow facilitates molecular crosstalk among tissues in a multiorgan device (Guzzardi et al., 2009). Reporting the cell density for an MPS in combination with the scaling factor and liquid volume will help assess its performance as a predictor of clinical outcomes.

For the purposes of proof-of-concept, *in vitro* organs are often made with cells from immortalized cell lines. Cell lines have the advantage of being highly reproducible and easy to access; however, compared to native cells, the cell signaling pathways and their metabolism may differ considerably due to cell line transformation (Zeilinger et al., 2016). The use of primary cells in early passages, i.e., with minimum number of subcultures done from the primary culture, helps limit this problem and create more realistic organ models.

The development of tissues using stem cells also provides a suitable alternative to using cell lines. Stem cell-derived tissues can be obtained from human adult, embryonic, or induced pluripotent stem cells (iPSCs). The cells can be seeded on a 3D matrix and allowed to undergo division and differentiation. The complexity and intricacy of a 3D model can limit the diffusion characteristics in the engineered tissue. Some of these limitations can be overcome by localizing the metabolically active cells to the periphery of the 3D model. McMurtrey (2016) outlines analytical models to determine the oxygen and nutrient diffusion in 3D constructs computationally.

A well-designed MPS must also be able to maintain cell viability during the entire length of an experiment. During the operation, the cellular functions of the involved tissues should be observed based on the study objectives. These may involve continued monitoring of enzymatic activity, and metabolite synthesis in liver cells, electrical activity in cardiac or neuronal cells, and barrier integrity in endothelial cells. A detailed record of “what” and “how” of each aspect of experimental design helps in understanding and reproducing any study.

### Fluid Flow Rates

When trying to replicate *in vivo* drug metabolism rates in an MPS, it is important to also consider flow rates and fluid residence times within each organ compartment. It has previously been proposed that the rate of drug absorption and conversion in the liver and in any other tissue is determined by the amount of time the blood surrogate spends inside the organ chamber (Eqs. 4-1, 4-2; Bruus, 2008). Therefore, *in vivo* perfusion rates per organ volume should be replicated in an MPS as well.

(4-1) $$t_{\text{res}}=\frac{V_{\text{o}}}{Q_{\text{o}}}$$

(4-2) $$\frac{\text{d}C_{\text{o}}}{\text{d}t}=\frac{C_{\text{b}}}{t_{\text{res}}}-\frac{C_{\text{o}}}{t_{\text{r}}P_{\text{o}}}-\frac{CL_{\text{int}}fC_{\text{o}}}{V_{\text{o}}}$$

Here, *t*_res_ is the residence time of blood flow in the organ in minutes, *V*_o_ is the volume of an organ in milliliters, *t* is the time in minutes, *C*_b_ is the concentration of drug in blood in mmol/mL *C*_o_ is the concentration of drug in the organ in mmol/mL, *Q*_o_ is the blood flow rate going through the organ in mL/min, *CL*_int_ is the intrinsic clearance of the drug in the organ in mL/s and *f* is the unbound fraction of drug.

When optimizing flow rates, the adequacy of oxygen supply to each tissue must also be carefully considered. Since cell culture medium has a limited capacity to carry oxygen throughout the system, a limited oxygen supply may also limit metabolic conversion rates. Faster flow rates can potentially alleviate this limitation. The cell culture medium should also be able to take up new oxygen and release carbon dioxide while recirculating, potentially through an open reservoir, or through breathable MPS construction materials. On the other hand, as discussed in section “MPS for Modeling the Immune System,” the shear generated by fluidic flow must not exceed the threshold at which it will cause damage to the cells cultured in the MPS. *In vivo*, most cells (other than endothelia or epithelia) are only subject to small shear stress derived from the interstitial flow of liquid. Hence, unless the cells of a tissue are protected by an epithelial cell layer or an endothelial cell layer, the shear resulting from the flow of cell culture medium inside an organ chamber is typically kept below 0.025 Pa (0.25 dynes/cm^2^) (Oleaga et al., 2016).

### Organ Chamber Arrangement and PBPK-Guided Designs

To create a multi-organ MPS, individual organ mimics are coupled together in a physiologically relevant manner. For example, an MPS that mimics the first-pass metabolism of orally taken drugs combines the GI-tract epithelium with liver tissue so that the drug must first pass through the epithelium before reaching the liver and the fluidic system that represents the body’s systemic circulation (Esch et al., 2014).

For more complex systems with more organ mimics, PBPK models are used as a guide to construct MPS (Sung et al., 2014, 2019). Using this method, organ chambers are connected to each other in a row or in parallel, depending on how they are connected in the body. As a basic principle, a PBPK model assumes the MPS to be a collection of interconnected compartments. Organ chamber connected via vascular channels provide pharmacodynamic information that individual organ-on-chip devices are unable to achieve. A semi-permeable membrane between vascular and endothelial cells facilitates drug transport and metabolism studies. A fluidically connected vascular network of organ chips enables the study of drug distribution in the organ. Studies show that linking multiple organ on chip systems by using scaling techniques can help advance pharmacokinetic and pharmacodynamic studies (Chang et al., 2017; Cyr et al., 2017).

An advantage of using PBPK models to design MPS is that such a model can also be created for the MPS itself. All design considerations, like organ chamber sizes and flow rates, are computationally entered into the model, and upon solving the model’s mass balance equations, the expected time-dependent drug concentration is obtained. Comparing computationally obtained drug and drug metabolite concentrations with concentrations obtained experimentally with the corresponding MPS can help verify any assumptions that were used to design the system, and gain confidence in the tissue responses that were observed. Adiwidjaja et al. (2020) implemented a PBPK model to determine optimal dosage and potential drug interactions with imatinib, a chronic myeloid leukemia drug, using *in vitro* drug metabolism and *in vivo* pharmacokinetic data. The model was then extrapolated to children by factoring in the change in organ size, volume and drug metabolism.

Due to the simplified representation of the human body when using *in vitro* MPS, extrapolation of information from *in vitro to in vivo* (IVIVE) system requires the use of mathematical models. One such example is the study conducted by Herland et al. (2020). These authors show adaptation of a computational model of the *in vitro* linked multi-organ chip to the pharmacokinetic/pharmacodynamic (PKPD) profiles of orally taken and injectable drugs observed in humans *in vivo* (Herland et al., 2020).

### Device Material

Certain considerations in selecting an MPS material include its cost, reusability, sterility, opacity, hydrophilicity, and breathability. There are a variety of biocompatible materials that have been used to make MPS. An integral part of designing a functioning tissue is the proper selection of biocompatible, non-toxic device materials. Since silicon is a material for which many microfabrication processes are available (especially deep reactive ion etching processes), some of the first devices were made from silicon and used with a glass or plastic cover (Sin et al., 2004; Tatosian and Shuler, 2009).

Silicon can also be used to create templates for casting silicone materials such as PDMS. PDMS has become one of the most used materials for MPS fabrication because it is inexpensive, and a single silicon template can be used to cast hundreds of PDMS devices. PDMS also has the advantage of being stretchable, a property that has been used successfully to re-create the mechanical stretching the lung epithelium experiences during the breathing process (Huh et al., 2010). However, when using PDMS, some drug loss can occur due to its affinity to hydrophobic compounds (Vernetti et al., 2017).

More recently, MPS have been made from 3D-printed polymers (Esch et al., 2016; Chen et al., 2020). Like PDMS devices, 3D-printed systems must be checked for drug and drug metabolite losses due to adsorption to device surfaces. Pre-coating of the material to reduce its drug binding properties can act as a potential solution. Parylene, a biocompatible polymer, coated on a 3D printed device does not absorb or react to drugs or metabolites. Esch et al. (2016) used parylene C to coat the 3D printed polymer of their device platform and two-organ chips. This system is primarily advantageous in cases where the drugs to be tested are hydrophobic and cannot be tested with PDMS systems due to adsorption issues.

Since cell culture is highly dependent on the physical and chemical properties of the environment, recreating the *in vivo* environment in an *in vitro* setup takes precedence. A recent development, 3D bioprinting of cell types and other materials provides increased control over the structural and cellular environment in a one-step process. Yi et al. (2019) designed a glioblastoma (GBM)-on-chip model using 3D bioprinting process to mimic the *in vivo* environmental cues of the disease. They used patient derived cancer cell-laden bioink along with silicon ink to print the cells onto a sterilized surface modified slide glass, creating a GBM-on-chip model. This approach helped Yi et al. (2019) achieve an anatomically similar spatial tissue organization of GBM *in vitro.* Combining the strengths of bioprinting with those of microfluidics can give rise to a physiologically realistic alternate to animal models. Fetah et al. (2019) provide a detailed review on the recent advancements in the use of bioprinting in organ-on-chip systems.

## Drug Toxicity Testing With MPS

To enable widespread adoption of MPS for drug toxicity screening, the devices need to be reliable, affordable and validated. Device validation often involves the use of a model drug whose toxic effects on the human body are known. While both single and multi-organ MPS can be used to detect any drug’s direct effects on tissues, it is a particular strength of multi-organ MPS to also uncover secondary toxicity from drug metabolites.

This was demonstrated by a study conducted by Sung et al. (2010) using the anticancer drug 5-fluorouracil (5-FU) in their experiments. *In vitro* testing of breast cancer drug, tamoxifen, with verapamil showed off-target effect on contractility, beat frequency and conduction velocity of cardiac cells by the metabolites of tamoxifen (McAleer et al., 2019a). Terfenedine, currently withdrawn antihistamine drug, is a commonly used cardiotoxin for proof-of-concept studies. A 28-day co-culture of hepatocytes and cardiomyocytes in MPS by Oleaga et al. (2018) was used to show that non-cardiotoxic drugs, terfenadine and cyclophosphamide, generate cardiotoxic metabolites upon liver transformation. *In vitro* experiments in another liver-heart MPS revealed that the toxic effects of terfenadine were dependent on the concentration of drug within the cardiomyocytes rather than the concentration in the media (McAleer et al., 2019b). Using the experimental drug response data, McAleer et al. (2019b) created a PKPD model that correctly predicted the pharmacokinetics of terfenadine and its metabolites in the MPS. Other model toxicants could be drugs that passed trials with animal models but showed toxicity in clinical trials with humans.

The interconnected organs in an MPS device provide an understanding of the fate of the drug in the body, and the collective response of the organs to the drug, thus creating an efficient model for toxicity studies. *In vitro* studies also help determine the potential experimental dose range of a drug or a combination of multiple drugs which can then be tested in a clinical setting.

MPS devices can also be used for drug testing in both the pre-clinical phase and post-release market phase. After the approval of a drug, complications such as failed phase 4 clinical trials, post-market failure, and black-box warnings can arise (Mol et al., 2013). Many commercially available drugs cause hepatotoxicity or cardiotoxicity, calling for the need to conduct comprehensive drug safety assessment even post-approval (Minotti, 2010). Drugs that passed clinical trials and then show failures post-approval are good candidates for proof-of-concept MPS studies that aim to uncover rare or longer-term effects. Database (AdisInsight; ClinicalTrials.gov, 2000) on safety analysis of developed drugs or drugs under development, drug toxicity profiles, and mechanisms of drug action can aid in designing experiments for MPS validation.

## Conclusion

The lengthy and expensive process of drug development often results in failed clinical trials with humans due to low efficacy or higher than expected toxicity, putting into question the reliability of animal testing as a method for drug evaluation.

The development of *in vitro* human body mimics is a challenging, but exciting goal with abundant research prospects. Multi-organ MPS present a promising route to improve outcome predictions of clinical trials. The devices are designed to be as physiologically realistic as possible by factoring in functional organ volumes, fluid flow rates, and fluid-to-cell ratios. In addition, regulating the blood surrogate volume to physiological levels is of utmost importance to obtain precise concentrations of drug metabolites in the blood surrogate volume. With the development of MPS, the scope of drug development is vast. Easy, reliable, and standardized models for drug testing can aid in development of personalized medicines as well. The use of patient-derived stem cells in MPS can provide patient-specific reactions to the drug or a combination of drugs.

The novelty of MPS also brings variability in functionality. Reporting of MPS design criteria such as scaling factor, fluid volumes, and cellular components used in the research can help standardize the process and make the use of MPS accurate and reproducible.

## Author Contributions

ME: conceptualization. MM and ME: writing – original draft. PF: writing – MPS for the immune system (section “MPS for Modeling the Immune System”). MM, GM, and ME: writing – review and editing. YY: mathematical equations and PBPK models (Figure 5). All authors contributed to the article and approved the submitted version.

## Author Disclaimer

^‡^Certain commercial entities, equipment or materials may be identified in this document to describe an experimental procedure or concept adequately. Such identification is not intended to imply recommendation or endorsement by the National Institute of Standards and Technology, nor is it intended to imply that the entities, materials, or equipment are necessarily the best available for the purpose.

## Conflict of Interest

The authors declare that the research was conducted in the absence of any commercial or financial relationships that could be construed as a potential conflict of interest.

## Publisher’s Note

All claims expressed in this article are solely those of the authors and do not necessarily represent those of their affiliated organizations, or those of the publisher, the editors and the reviewers. Any product that may be evaluated in this article, or claim that may be made by its manufacturer, is not guaranteed or endorsed by the publisher.
